# Integrated Chronology, Flora and Faunas, and Paleoecology of the Alajuela Formation, Late Miocene of Panama

**DOI:** 10.1371/journal.pone.0170300

**Published:** 2017-01-20

**Authors:** Bruce J. MacFadden, Douglas S. Jones, Nathan A. Jud, Jorge W. Moreno-Bernal, Gary S. Morgan, Roger W. Portell, Victor J. Perez, Sean M. Moran, Aaron R. Wood

**Affiliations:** 1 Florida Museum of Natural History, University of Florida, Gainesville, Florida, United States of America; 2 Smithsonian Tropical Research Institute, Ancon, Panama; 3 New Mexico Museum of Natural History, Albuquerque, New Mexico, United States of America; 4 Department of Geological Sciences, University of Florida, Gainesville, Florida, United States of America; 5 Department of Biology, University of Florida, Gainesville, Florida, United States of America; 6 Department of Geological & Atmospheric Sciences, Iowa State University, Ames, Iowa, United States of America; Institute of Botany, CHINA

## Abstract

The late Miocene was an important time to understand the geological, climatic, and biotic evolution of the ancient New World tropics and the context for the Great American Biotic Interchange (GABI). Despite this importance, upper Miocene deposits containing diverse faunas and floras and their associated geological context are rare in Central America. We present an integrated study of the geological and paleontological context and age of a new locality from Lago Alajuela in northern Panama (Caribbean side) containing late Miocene marine and terrestrial fossils (plants, invertebrates, and vertebrates) from the Alajuela Formation. These taxa indicate predominantly estuarine and shallow marine paleoenvironments, along with terrestrial influences based on the occurrence of land mammals. Sr-isotope ratio analyses of *in situ* scallop shells indicate an age for the Alajuela Formation of 9.77 ± 0.22 Ma, which also equates to a latest Clarendonian (Cl3) North American Land Mammal Age. Along with the roughly contemporaneous late Miocene Gatun and Lago Bayano faunas in Panama, we now have the opportunity to reconstruct the dynamics of the Central America seaway that existed before final closure coincident with formation of the Isthmus of Panama.

## Introduction

The late Miocene was an important time to understand the biodiversity dynamics in the Neotropics. No place within this region was more important than Panama because it functioned as both a gateway and barrier to, respectively, interoceanic and intercontinental dispersal of Neotropical biotas. Despite this importance, until recently our knowledge of late Miocene biotas has been restricted to marine faunas such as the hyperdiverse, Gatun Formation, which preserves an excellent record of both invertebrates [1] and chondrichthyans [2]. In contrast to the marine record, up to now no terrestrial macrofossils have been reported in Panama from the time interval between the early Miocene of the Panama Canal localities [3] and the late Pleistocene of the Azuero Peninsula [4]. The apparent lack of terrestrial sedimentation, as evidenced by the paucity of outcrops during the late Miocene portion of this interval, likely resulted from the rapid uplift and consequent erosion during the formation of the isthmus [5]. This hiatus of almost 19 million years in the non-marine fossil record of Panama is nevertheless critical to understanding the terrestrial faunal dynamics before and after the final closure of the Central American Seaway and rise of the Isthmus during the Pliocene.

The Miocene proboscidean *Gomphotherium*, which was recently described from Lago Alajuela (previously Lake Madden), begins to fill in this critical hiatus [6]. As it is known from elsewhere in Holarctica [7], the genus *Gomphotherium* has a fairly long temporal range, between about 15 to 5 Ma, and it is this interval that we have previously assumed for the age of the Alajuela Formation. Over the past two years we have discovered the original site where *Gomphotherium* was collected in 1959 and conducted further field work to recover additional fossils from the sedimentary units that crop out along the shore and islands of Lago Alajuela, particularly during times of lowered lake level.

The purpose of this report is to present a preliminary description of the invertebrate and vertebrate faunas and floral remains collected from Lago Alajuela, describe the physical stratigraphy, present new Sr-isotope ratio age determinations, and discuss the significance of these data in light of our understanding of the biodiversity and paleobiogeography of the late Miocene of Panama. We realize that certain aspects of this paper are incomplete, most notably the faunas, which still represent only a small fraction of the overall ancient biodiversity. Nevertheless, the implications of these occurrences, plus the new Sr-isotope dates, have considerable significance for an area that is of great current interest in terms of New World tropical (paleo)biology.

### General Background and Previous Geological Studies

**Fig 1:**
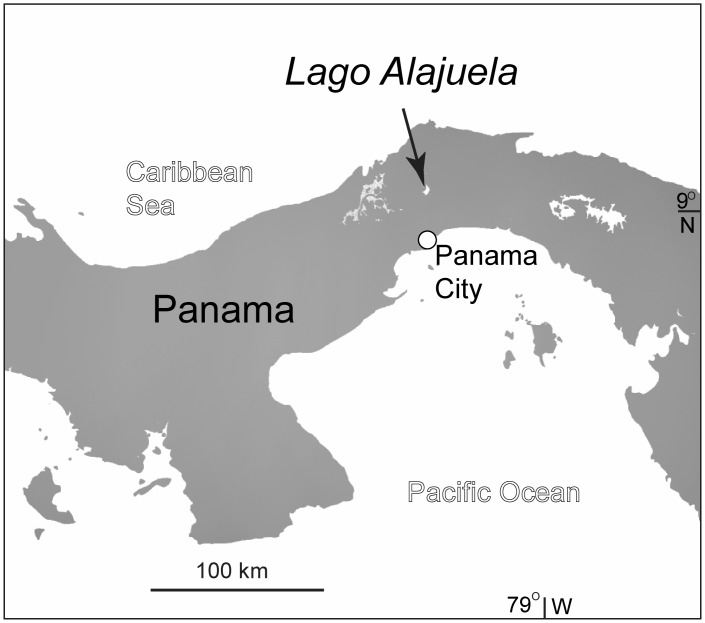
General location of Lago Alajuela in northern Panama.

**Fig 2:**
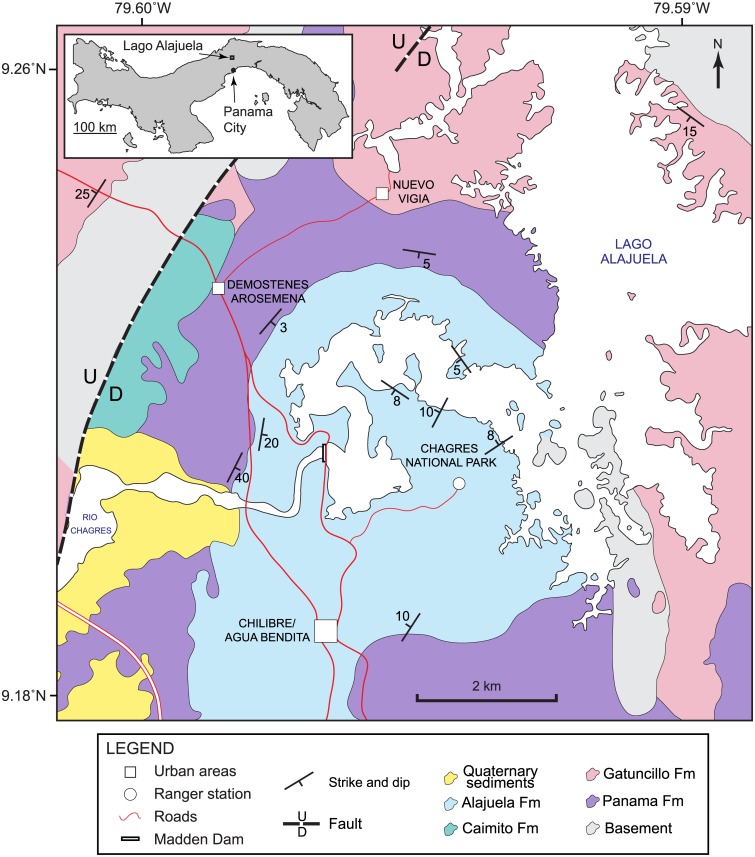
Geological map of Lago Alajuela region, modified from Woodring et al. [11].

Sediments referred to the Alajuela Formation were deposited in the Panama tectonic basin [8] and crop out along the shore of Lago Alajuela in the Panama Province (Fig 1) at a general lat. 9.2124° N, long. 79.5936° W and an elevation of about 80 m. The fossil localities, including the initial *Gomphotherium* discovery in 1959 by John Turner, occur along the shores and adjacent small islands of the southwestern portion of the lake (Table 1), less than 1 km from the Chagres National Park Headquarters, which is also the general site of the now defunct Madden Lake Boy Scout Camp [9]. Woodring [10] mapped relevant outcrops along the shore of Lake Madden as pertaining to the lower to middle Miocene Caimito Formation (including the Alajuela Member), whereas Woodring et al. [11] mapped these as the upper member of the upper middle Miocene Alajuela Formation, consisting of tuffaceous sandstones, calcareous sandstones, and limestones. In these maps, the Alajuela Formation is depicted (in the legend) to underlie the well-known Gatun Formation, although the direct superpositional field relations to confirm these relative ages are not well supported. In fact, the Sr-ratio age determinations presented here address the relative ages of these formations, as will be discussed below. Our geological map, modified from Woodring et al. [11], is presented in Fig 2.

**Table 1:** List of location sites from which the fossils described in this report were collected from the upper Miocene Alajuela Formation, Lago Alajuela, Panama (UF VP general locality YPA088, lat. 9.2124°, long. -79.5936°.

Site Designation*	Latitude (°)	Longitude (°)
630041	9.2159	-79.5972
630042	9.2156	-79.595
630043	9.2141	-79.5942
630044	9.2130	-79.5932
630045	9.2149	-79.5946
630046	9.2223	-79.5978
630048	9.2119	-79.5929
630049	9.2114	-79.5919
630050	9.2200	-79.5888
630051	9.2107	-79.5915
630052	9.2090	-79.5913
630053	9.2164	-79.6012
630061	9.2160	-79.5983
630066	9.2161	-79.5987

**Fig 3:**
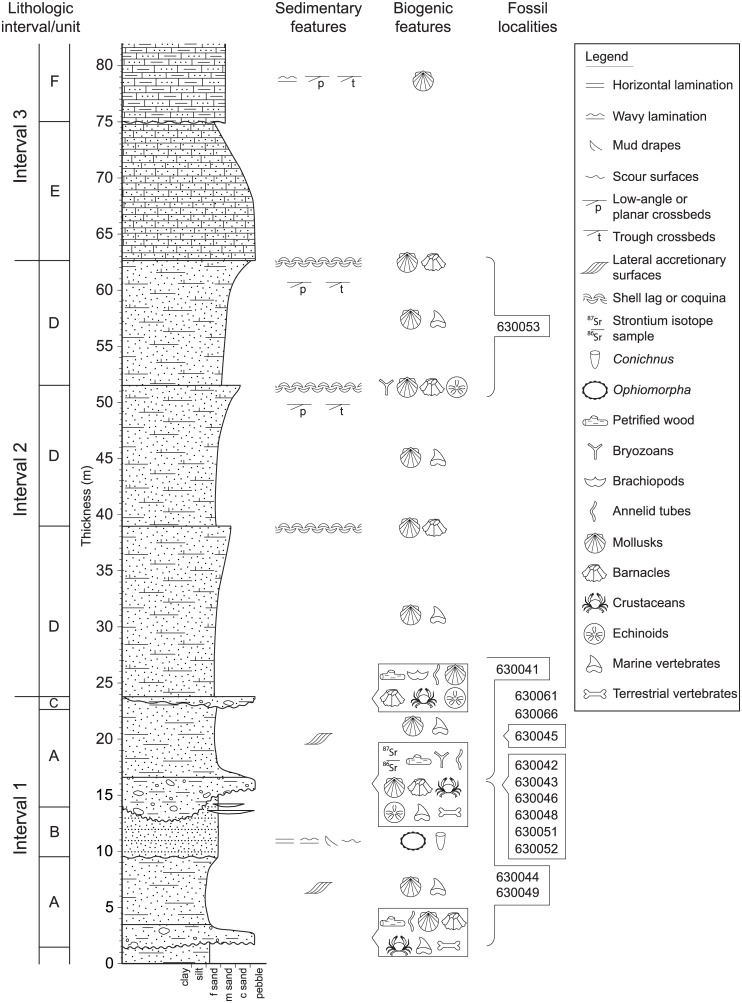
Composite lithostratigraphic section of the Alajuela Formation as exposed in northeastern Chagres National Park, Panama along Lago Alajeula. Key lithologic intervals and units described in the text are marked to the left. Relevant fossil localities and sedimentary and biogenic features are also shown. No well-exposed formational contacts between the Alajuela Formation and underlying and overlying formations occur in the study area. Consequently, the 82 m of section shown here represent only a fraction of the total thickness of the Alajuela Formation.

*The relative stratigraphic positions of these sites are also listed on Fig 3.

## Materials and Methods

This study integrates three domains of investigation: i.e., lithostratigraphy, Sr-isotope dating, and paleontology. The lithostratigraphic work was done in the field by measuring and describing stratigraphic sections and locating these via GPS. With regard to Sr-ratio dating, fossils were collected from our composite measured section as documented below. Powdered low-magnesium calcite samples were drilled from the interior of each specimen using a hand-held Dremel tool with a carbide dental burr. Approximately 0.01 to 0.03 g of powder was recovered from each fossil sample and these were analyzed according to standard techniques [12]. The powdered samples were dissolved in 100μl of 3.5 N HNO_3_ and then loaded onto cation exchange columns packed with strontium-selective crown ether resin (Eichrom Technologies, Inc.) to separate Sr from other ions. Sr isotope analyses were performed on a Micromass Sector 54 Thermal Ionization Mass Spectrometer equipped with seven Faraday collectors and one Daly detector in the Department of Geological Sciences at the University of Florida. Sr was loaded onto oxidized tungsten single filaments and run in triple collector dynamic mode. Data were acquired at a beam intensity of about 1.5 V for ^88^Sr, with corrections for instrumental discrimination made assuming ^86^Sr/^88^Sr = 0.1194. Errors in measured ^87^Sr/^86^Sr are better than ±0.00002 (2σ), based on long-term reproducibility of NIST 987 (^87^Sr/^86^Sr– 0.71024). Age estimates were determined using the Miocene portion of Look-Up Table Version 4:08/03 associated with the Sr isotopic age model [13].

With regard to paleontology, invertebrate, plant, and vertebrate fossils were collected from the individual measured sections and integrated into the composite section described below. Fossils were collected under permit 2014–21 issued by the Ministerio de Comercio e Industries, Dirección Nacional de Recursos Minerales, Republic of Panama. Fossils were collected mostly by surface prospecting; although matrix was collected in an attempt to recover microfauna, this proved unsuccessful. The identification of some invertebrate fossils was enhanced by making casts of some moldic mollusks. Fossils were trimmed with a rock saw, cleaned, and a room temperature vulcanizing (RTV) silicone rubber was vacuumed into the external molds. Once cured (12 hours), the silicone rubber peels were removed for examination and later identification. In order to study fossil wood specimens, thin-sections were produced using standard techniques [14] along transverse, tangential, and radial planes. A total of 41 specimens were examined in transverse section to evaluate the quality of preservation and to distinguish palms from dicots. Twenty-four of dicot woods were sectioned along tangential and radial planes for further identification. We used the InsideWood Database [15, 16] and Metcalfe and Chalk [17] to identify the fossils to families.

The fossils described below are contained within three paleontological collections at the Florida Museum of Natural History, University of Florida, and are referred to by the acronyms UF-I (Invertebrate Paleontology), UF-P (Paleobotany), and UF-V (Vertebrate Paleontology). The relevant data for these can be retrieved on-line at www.flmnh.ufl.edu.

## Litho- and Biostratigraphy of the Alajuela Formation

The Alajuela Formation includes a >25 m-thick basal package of interbedded, clast-supported conglomerates and litharenite sandstones that grades into an ~85 m-thick package of calcareous sandstones and calcarenites, representing a transition from tide-dominated, potentially estuarine, coastal environments to wave-dominated, shallow-water carbonate environments [10,18]. The 82 m-thick composite section (Fig 3), measured in proximity to the fossil localities on the southern extent of Lago Alajuela (Table 1), is subdivided into three distinctive lithological intervals to summarize major facies transitions during this transgression.

Within the basal-most Interval 1, laterally-extensive horizons of amalgamated conglomerate lenses fine upwards into fine-medium grained sandstones (Unit A of Fig 3), truncated by erosional contacts with overlying units. The best exposed amalgamated conglomerate (near the 15 m level in the composite section) exhibits substantial variability in thickness (1–6 m) with an average thickness of ~3 m. The amalgamated conglomerates are generally clast-supported, but locally matrix-supported, with the coarse grain fraction rarely exceeding 5 cm in diameter. Weathered exposures of the conglomeratic horizons erroneously appear matrix-supported due to diagenetic dissolution of aragonitic shell material. Prior to such dissolution, the coarse fraction would likely have been dominated by mollusk shells, whereas well-rounded, pebble- to cobble-sized volcanic fragments (welded tuff and andesite) and silicified woods comprise the minor component of the coarse fraction. Fossil invertebrate remains primarily consist of internal and external molds of mollusk shells preserved in fine-grained sand matrix as well as some original calcitic shell material of scallops and oysters (also see discussion below). The amalgamated conglomerates contain the most abundant vertebrate fossils, both well-preserved remains of marine vertebrates (e.g., sharks) and highly weathered remains of terrestrial vertebrates.

The conglomerate exhibits a dm-scale gradational contact with a poorly sorted, fine-medium grained sandstone with abundant lithic and feldspar grains. The sandstone is locally tuffaceous and exhibits, in some exposures, low-angle, dm-thick bedforms that are internally massive and dip perpendicularly to the overall attitude of the Alajuela Formation. Otherwise, the sandstone appears massive and highly bioturbated, containing a lower density of molluscan molds than the underlying conglomerate. Original molluscan shell material and marine vertebrate fossils are present but rare. No terrestrial vertebrate remains have been found within the sandstone horizons of Unit A.

**Fig 4:**
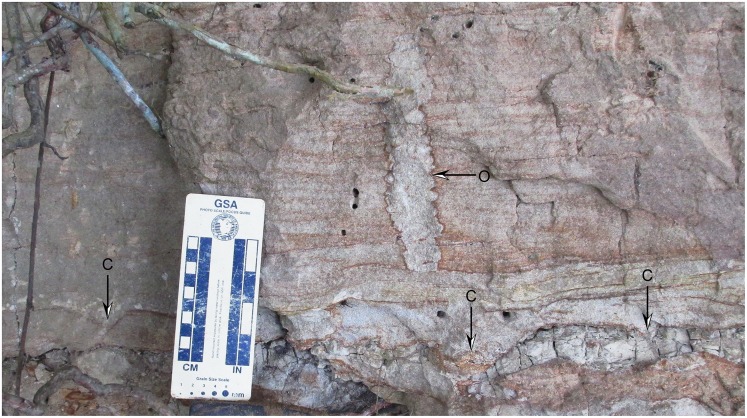
Ichnofossils of *Conichnus* (C symbol) and *Ophiomorpha* (O symbol) from Interval 1 of the Alajuela Formation.

One exposure of Interval 1, within the 9–14 m levels of the composite section, includes a moderately sorted, fine-medium grained tuffaceous sandstone with distinctive mm- to cm-thick horizontal bedding and wavy laminations (Unit B of Fig 3). Scour and fill structures with dm-scale widths and occasional mud drapes are present near the erosional contact with the underlying Unit A lithologies. No body fossils are present in this unit, but rare and well-preserved ichnofossils, primarily *Conichnus* and vertically-oriented *Ophiomorpha* (Fig 4), are preserved that cut across and deform bedding horizons. The infilling of these burrows consists of the same fine tuffaceous sand of overlying horizons.

The base of Interval 2 exhibits a highly irregular, erosional contact with either a poorly sorted, bioturbated sandstone of Unit A lithologies or a laterally discontinuous unit of clast-supported conglomerate (Unit C in Fig 3). This interval typically occurs well above lake levels in the study area and is covered by vegetation. Consequently, continuous fresh exposures exhibiting diagnostic sedimentary structures are relatively rare. The dominant lithology in Interval 2 appears to be a well-cemented, fine-grained litharenite that coarsens upwards into a medium-grained sand capped by a shell lag horizon of fragmented bivalves and gastropods (Unit D of Fig 3). The density of shell fragments at the top of the coarsening-upwards sequences locally approaches that of a coquina. A minimum of three coarsening-upward sequences is preserved in Interval 2, and although the litharenite appears massive in most exposures, trough cross-bedding and low-angle planar cross-bedding are evident locally.

The base of Interval 3 is marked by a cm-scale gradational contact between an underlying shell lag in Interval 2 and an overlying fine-grained calcareous sandstone with lithics and occasional trough cross-bedding and ripple marks (Unit E of Fig 3). A calcarenite occasionally interbedded with sandy limestone (Unit F of Fig 3) occurs stratigraphically above, separated from the underlying calcareous sandstone by an irregular, erosional contact. Sedimentary structures vary within the calcarenite from trough cross-bedding to wavy bedding to low-angle planar cross-bedding, suggesting substantial changes in flow velocities at the time of deposition. The lithologies in Unit F were originally described by Woodring [10] as the Alhajuela [sic.] Sandstone Member of the Caimito Formation. However, based on the age constraints presented below for the Alajuela Formation, this attribution to the late Oligocene—early Miocene Caimito Formation is no longer supported. The stratigraphic thickness of Interval 3 was not measured in the present study but was reported by Woodring [10] as being approximately 85 m.

Interval 1 is tentatively interpreted as sediment deposited in the distal area of a tide-dominated estuary. The amalgamated conglomerate horizons with variable thickness likely represent lag deposits within tidal channels whereas the overlying bioturbated sandstones represent tidal sand bars with lateral accretionary surfaces (i.e., the low-angle, dm-thick bedforms described above in Unit A). It should be noted that we have no unequivocal evidence for net landward movement of sediment, a defining characteristic of estuarine deposits in the sedimentary record [19], other than the overall transgressive sequence in the Alajuela Formation. However, the transition from 1) channelized conglomerates to 2) laterally-accreting sand bodies to 3) horizontally-bedded well-sorted sand, all with marine body and trace fossils, is consistent with a transition from outer estuarine sand bars to upper flow regime sand flats in the tide-dominated estuary model of Dalrymple et al. [19]. The association of horizontal bedding, cut and fill structures, and *Ophiomorpha* and *Conichnus* ichnofossils within Interval 1 furthers supports an estuarine depositional environment [20].

Deposits within Intervals 2 and 3 in the composite section are interpreted to have been deposited in wave-dominated nearshore environments. A precise depositional interpretation for Interval 2 lithologies, however, is problematic without well-exposed diagnostic sedimentary structures. The presence of fine to medium grained litharenites with some evidence of trough cross-bedding and low-angle planar cross-bedding tentatively suggests an upper to middle shoreface depositional environment, but better exposures on the northern shore afforded by a drop in lake levels are necessary to verify this interpretation. The Interval 2 to 3 transition is marked by an increase in carbonate material that culminates in a well-sorted calcarenite, indicating a shift from terrestrially-sourced sediment to marine-derived sediment without a concurrent increase in water depth.

Most of our fossil samples have been collected from outcrops of the Alajuela Formation along the southwestern shores of Lago Alajuela. These localities are georeferenced and can be placed stratigraphically within our composite measured section (Fig 3). In addition to these localities, however, several other fossil localities from outcrops that appear to be correlated to the Alajuela Formation were sampled from Isla Vigia and other smaller islands within a few km west of our stratigraphically controlled localities. These localities yielded fossil invertebrates, wood (*Parinarioxylon* sp.), and vertebrates including turtles and several taxa of shark teeth, dominated by the diagnostic *Carcharocles megalodon*. We realize that although the island localities are not stratigraphically calibrated like those on shore, they nevertheless attest to the additional paleontological potential of the Alajuela Formation.

## Sr-isotope Ratio Age Determinations

So far as lithostratigraphic studies in the field demonstrate, no suitable volcanic units crop out within our measured sections of the Alajuela Formation. The occurrence of original shell material from marine fossils therefore provided an opportunity to determine the age of the unit using ^87^Sr/^86^Sr geochronology. Mollusk shells (scallops and oysters) were collected from a ~2 m thick interval about 10 to 12 m above the base of the composite section and below a prominent fossiliferous conglomerate (Fig 3). As will become important below in the discussion with respect to the age of diagnostic fossils, this zone also approximates the levels where the terrestrial vertebrates were collected.

**Fig 5:**
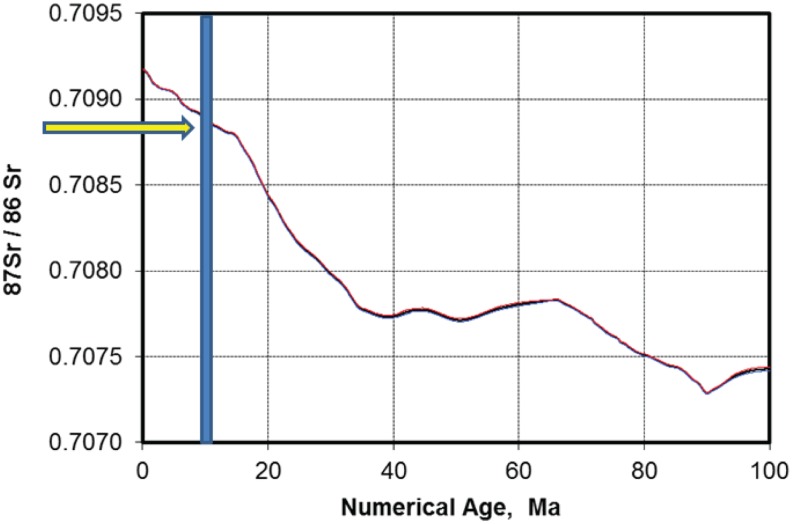
The ^87^Sr/^86^Sr ratios of eight shells analyzed from the Alajuela Formation yielding an age of 9.77 ± 0.22 Ma based on interpolation from McArthur et al. [16].

With the exception of sample LKA-1, which is interpreted to represent an outlier due to diagenetic alteration observed in shell cross-sections, ratios were tightly clustered between 0.708850 and 0.708920 (Table 2). Comparison of the mean ratio with the global seawater ^87^Sr/^86^Sr curve for the Neogene [13] indicates a late Miocene age of 9.77 ± 0.22 Ma for this portion of the Alajuela Formation (Fig 5). The strontium dates also confirm a late Miocene age for the associated vertebrate fossils including the biochronologically informative horses and proboscidean *Gomphotherium* sp.

**Table 2:** ^87^Sr/^86^Sr ratios and age determinations based on the eight samples analyzed from the Alajuela Formation, Panama. These samples were collected from locality 630044 (9.2130°, −79.5932°; Table 1).

Sample	^87^Sr/^86^Sr	95%CI (U)	Age (Ma)	95% CI (L)
LKA-1	0.708714	15.99	16.09	16.19*
LKA-2	0.708882	9.93	10.12	10.34
LKA-3	0.708899	9.42	9.64	9.82
LKA-4	0.708909	9.04	9.33	9.54
LKA-5	0.708893	9.62	9.81	9.99
LKA-6	0.708850	10.98	11.22	11.54
LKA-7	0.708920	8.50	8.84	9.17
LKA-8	0.708905	9.20	9.46	9.66
MEAN (2–8)	0.708894	9.53	9.77	10.00

*Interpreted to be altered and therefore an analytical outlier.

## Paleobotany

Silicified woods occur as rounded to subangular clasts with low sphericity that often occur as float. These clasts tend to be concentrated in the lower part of the section and occasionally found as part of the coarse-fraction in Interval 1 (Fig 3). Based on 60 samples that we collected, the mean wood clast length is 7.3 cm (*s* = 2.4 cm) with an observed range of 3 cm to 29 cm. (Rounded chert clasts up to about 4 cm long also occur within the formation.)

**Fig 6:**
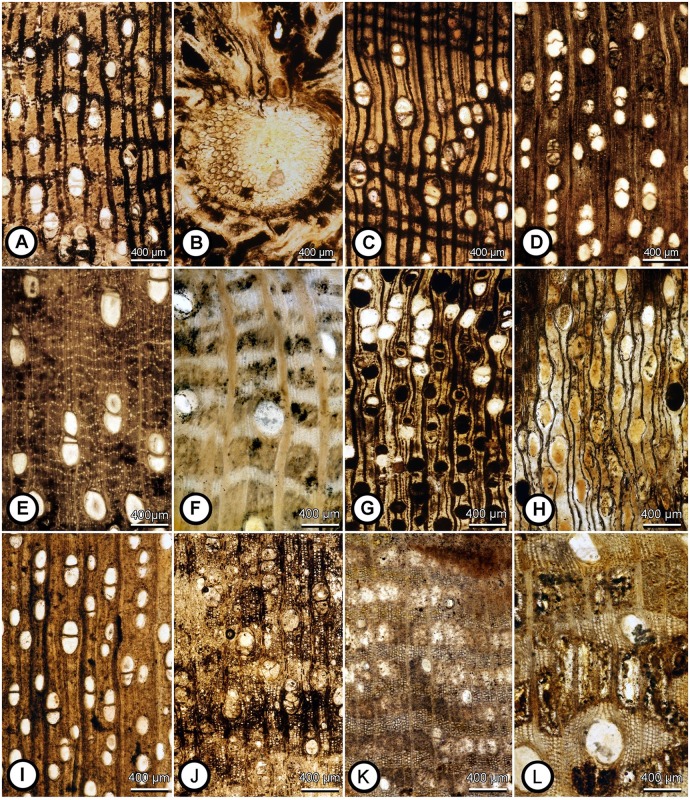
Cross sections of selected woods from the Alajuela Formation showing the variation in vessel diameter, vessel frequency, and conformations of axial parenchyma (e.g., narrow bands, absent/rare, uniseriate bands, wide bands, aliform/confluent). A. UF-P 63102 Malvaceae type a; B. UF-P 63103 Arecaceae; C. UF-P 63104 Malvaceae type b; D. UF-P 63105 Salicaceae/Rubiaceae; E. UF-P 63106 undet. angiosperm; F. UF-P 63109 Annonaceae; G. UF-P 63112 undetermined angiosperm; H. UF-P 63124? Phyllanthaceae; I. UF-P 63133 *Humiriaceoxylon* sp. (Humiriaceae); J. UF-P 63134 Sapindaceae; K. UF-P 63135 *Ficus* sp. (Moraceae); and L. UF-P 63146 Fabaceae.

Three of the 41 specimens examined in transverse section are palms, including at least two types: a one-vessel palm and a two-vessel palm. Although the assemblage is very rich, none of the dicot wood types (species) are represented by more than one specimen. A complete description and taxonomic allocation of each wood type is beyond the intended scope of this report; however, at this point some of the specimens can be identified to family level (Fig 6). Based on the characters preserved so far, the families recognized are Annonaceae, Phyllanthaceae, Fabaceae, Humiriaceae, Malvaceae, and Sapindaceae (Fig 6; S1 Table). Chrysobalanaceae is also represented in the Alajuela Formation, but from a different locality (630050) of unknown stratigraphic position relative to our measured sections. Each of these families is found in tropical forests in Panama today [21, 22]. All of the specimens are diffuse-porous, and only two (UF-P 63106 and UF-P 63120) have indistinct growth rings marked by narrow, thick-walled fibers, that may be annual based on the width and complacency. Some of the dicot woods examined in transverse section (six out of 38) have very large (>200 μm) mean vessel diameters, a character state typical of large canopy trees in tropical forests [23–25]. Most of the woods examined in longitudinal section (22 out of 24) have simple perforation plates and many of the woods (11 out of 38) have elaborate conformations of axial parenchyma (e.g., aliform, confluent, or wide bands). The rarity of growth rings, the presence of very large mean vessel diameters, abundant axial parenchyma, and the presence of both one- and two-vessel palms are all features found in taxa typical of Neotropical moist forests and rainforests [23, 24, 26–28].

The rounded, apparently rolled and abraded nature of the silicified woods in the conglomerates and sandstones of the Alajuela Formation raises the question whether these fossils represent silicic clasts that were reworked from (an) earlier depositional episode(s), or were contemporaneous with the vertebrate fauna, and transported as float before becoming waterlogged, sinking, and then replaced with silica. In a manner similar to the recent comparison of Panama fossils [29], 77 rare earth element (REEs) samples were analyzed and compared for the invertebrate, vertebrate, and wood fossils and sediments from the Alajuela Formation. In addition, we analyzed fossil woods from the lower Miocene Cucaracha Formation in the southern Panama Canal Basin [3, 30, 31] and from the Ocú area on the Azuero Peninsula, Panama [32]. Typically, when taphonomically mixed fossils form during different sedimentary cycles, they demonstrate different REE patterns [33] that allow temporal discrimination. This, however, was not the case in our analyses, and the REE results were equivocal, i.e., they did not discriminate fossils within the Alajuela Formation as well as in comparison with the Cucaracha Formation and Ocú area woods. Although we present the REE data in S1 Table, we do not report these within the text because of the ambiguous outcome of the analyses.

Regardless of the ambiguous results of the REE data with regard to the age of the fossils, other information can be brought to bear with regard to the Alajuela Formation woods. Autochthonous and parautochthonous assemblages of fossil wood preserved as charcoal and calcium-carbonate permineralizations from the Cucaracha Formation have lower species richness and higher evenness than the Alajuela Formation assemblage; but the same families are present in both units [30, 31, 34, 35], as well as older Miocene palynofloras [36–39]. The similarity of forest composition leaves open the possibility that the woods were reworked from lower Miocene deposits. On the other hand, the rarity of other clasts as large as the fossil woods in the conglomerates and coarse to medium sandstones of Interval 1 suggests the possibility that there may not have been enough energy for transporting relatively large silicified wood clasts. This therefore supports the hypothesis that the woods were transported as float prior to silicification and represent the vegetation roughly contemporaneous with the co-occurring fauna.

In summary, it is unclear whether the obviously rolled and abraded wood specimens were formed at the same time as the other fossils collected from the Alajuela Formation; however, their original age before reworking is likely constrained between about 21 Ma [40] and ~9.8 Ma (age of the Alajuela Formation; see above). Considering all the available evidence, we hypothesize that they are most likely to be of late Miocene age. Thus, these woods provide a glimpse of the forest community during the Miocene in the New World tropics, and help to demonstrate the continuity of tropical forest communities in Panama since that time.

## Invertebrate Fauna

**Fig 7:**
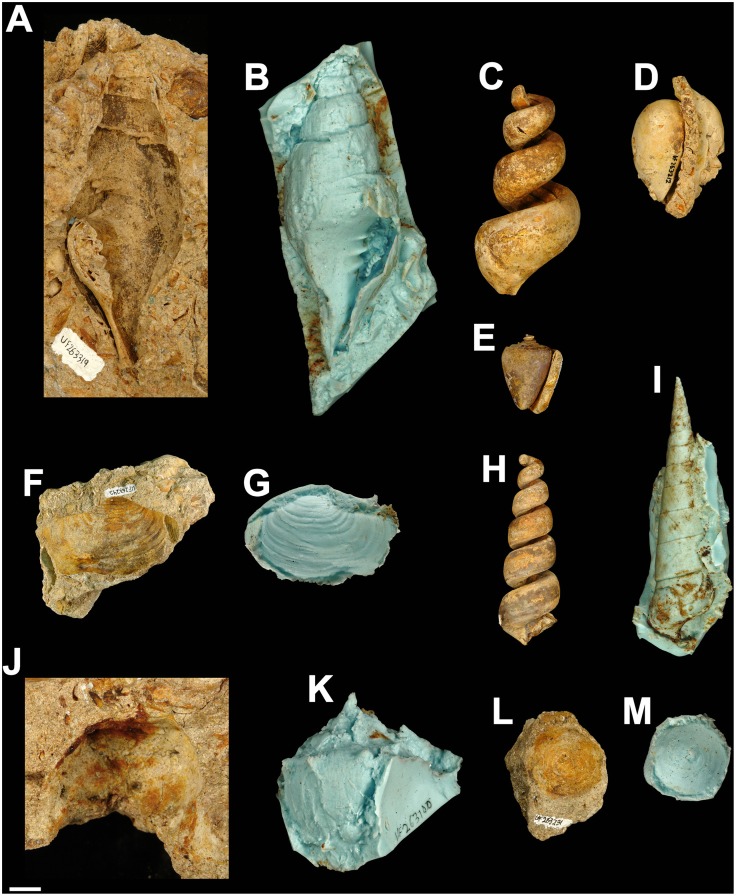
Selected examples of late Miocene moldic mollusks (originally aragonitic-shelled) from the Alajuela Formation, Lago Alajuela, Panama. Room temperature vulcanizing (RTV) silicone rubber was vacuumed into external molds to provide peels in order to facilitate identification. A-C. UF-I 263319, *Turbinella* sp., A. external mold—apertural view, B. peel, C. internal mold. D. UF-I 263212, Cypraeidae, internal mold—apertural view. E. UF-I 263119, *Conus* sp., internal mold—apertural view. F-G. UF-I 263242, *Panopea* sp., F. external mold, G. peel. H-I. UF-I 264011, Turritellidae, H. internal mold—apertural view, I. peel. J-K. UF-I 263100, *Melongena* sp., J. external mold—dorsal view, K. peel. L-M. UF-I 263231, *Calyptraea* sp., L. external mold—dorsal view, M. peel. Scale bar = 1 cm.

**Fig 8:**
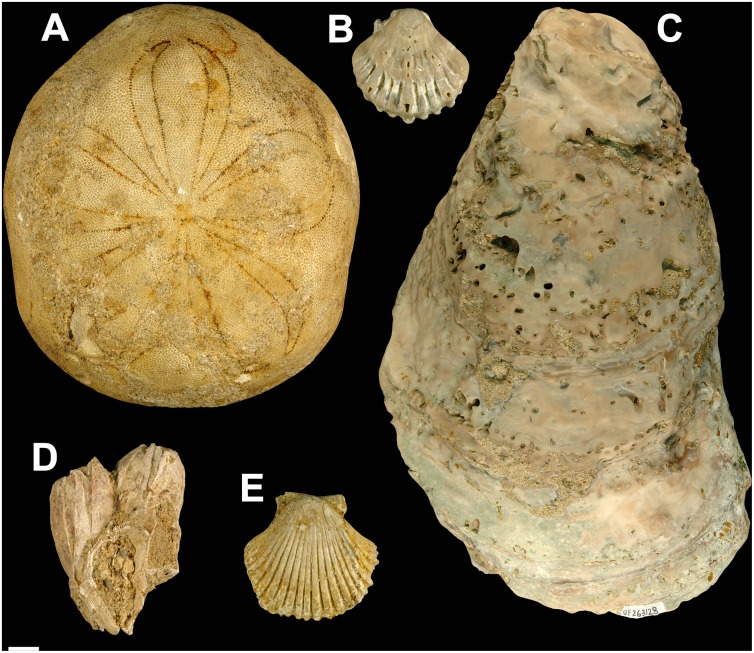
Selected examples of late Miocene calcitic-shelled fossils from the Alajuela Formation, Lago Alajuela, Panama. A. UF-I 261132, *Clypeaster* sp., test. B. UF-I 263343, *Nodipecten* sp., valve, C. UF-I 263128, *Crassostrea* sp., valve. D. UF-I 261080, cf. *Tamiosoma* sp., shell cluster. E. UF-I 261129, *Euvola* sp. valve. Scale bar = 1 cm.

Two types of preservation commonly occur in the Alajuela Formation invertebrate fossils from Lago Alajuela: 1) body fossils whose hard parts originally consisted of calcite (e.g., bryozoans, scallops, oysters, decapods, and echinoderms) and 2) more commonly, internal and external molds of organisms whose hard parts originally consisted of aragonite (corals and the majority of mollusks). Representative photographs of mollusks, arthropods, and echinoderms are presented in Figs 7 and 8.

### Phylum Cnidaria

Scleractinian corals are conspicuously absent from all localities indicating they were probably not present in this area at the time of deposition. Corals are typically found in normal marine conditions but do not tolerate the influx of freshwater.

### Phylum Bryozoa

Only six lots of cheilostome and two lots of cyclostome bryozoans were recovered from the deposits of Lago Alajuela. Most were poorly preserved, either leached or in one case moldic. All are encrusting forms; no erect forms were present. The majority of zoaria were collected from locality 630043 and these consisted of an indeterminate cyclostome (UF-I 261059), as well as one confidently identified to the Family Plagioeciidae (UF-I 261079). An unidentified cheilostome (UF-I 261057) and three lots of the genus *Chaperia* (UF-I 261058, 261060, 261061) were found as well. One specimen (UF-I 261122) was collected at locality 630053 and, although highly-weathered, was determined to be *Hippoporidra* sp. One moldic cheilostome (UF-I 263314) was found at locality 630048.

### Phylum Brachiopoda

One genus of brachiopod was discovered in the Alajuela Formation sediments along Lago Alajuela; the lingulid *Glottidia* sp. (UF-I 264953). The exposed valves are small (≤ 11 mm) and all were collected *in situ* at locality 630066. Living *Glottidia* is commonly observed in tropical and subtropical marine environments in intertidal to shallow subtidal zones [41], although Emig [42] suggests the intertidal zone is not the most optimal habitat for the genus because it is quite intolerant of lower salinity from freshwater influx.

### Phylum Annelida

Small mineralized tube fragments of polychaete worms (e.g., UF-I 263086) were collected from Alajuela Formation sediments at locality 630041 and occasionally found attached to calcitic skeletal remains of other invertebrates, mostly mollusks, from other sites.

### Phylum Mollusca

Four classes of mollusks (Bivalvia, Scaphopoda, Gastropoda, and Cephalopoda) are represented in the 218 specimen lots collected from seven Alajuela Formation localities (630041, 630043, 630044, 630048, 630049, 630053, and 630061). Of these, nearly 75% are bivalves, and approximately 25% are gastropods. Additionally, one scaphopod and one cephalopod taxon were recovered (see Table 2).

Interestingly, although much more abundant in terms of numbers of specimens, the bivalves are represented by only 13 families while the less abundant gastropods are represented by 19 families (most families based on just one or a few specimens). Common bivalve families represented in the Alajuela deposits are typically infaunal and include the Carditidae, Cardiidae, Veneridae, Corbulidae, and Hiatellidae (i.e., genus *Panopea*). Most are suspension feeding burrowers that inhabit shallow marine or estuarine environments. Common epifaunal families include Flemingostreidae (i.e., genus *Crassostrea*) and Pectinidae. At locality 630041, a large, well-developed *Crassostrea* oyster bar is preserved *in situ*. These *Crassostrea* valves are typically very large (up to 24 cm) and moderately to highly eroded. Some specimens resemble modern *Crassostrea gigas* (Giant Pacific Oyster). Additionally, at locality 630053 only calcitic body fossils of the bivalve families Plicatulidae (i.e., genus *Plicatula*) and Pectinidae (e.g., genera *Euvola* and *Nodipecten*) were recovered; no moldic material was found.

A single, recrystallized scaphopod (tusk shell) was recovered from locality 630044. Scaphopods are infaunal predators.

By far the most common gastropod family from the Alajuela deposits is Calyptreidae (i.e., genus *Calyptraea*). *Calyptraea*, recovered from localities 630043, 630044, 630048 and 630050, today inhabits near-shore, shallow- marine environments. Numerous other gastropod families (18) are represented in localities of the Alajuela Formation mentioned above (see Table 2). However, most are very rare.

From picked sediments of locality 630041, two guard-like sheaths (the calcareous covering that envelops all or part of the phragmocone) of coleoid cephalopods were also discovered (UF-I 264446 and UF-I 263088). This is the first report of fossil coleoids from the Cenozoic of Panama.

### Phylum Arthropoda

Acorn barnacle (Family Balanidae) shells and isolated opercular plates from at least two genera were observed in many of the Alajuela Formation outcrops along the lake. Collections from six localities (630041, 630043, 630044, 630048, 630049, and 630053) yielded 39 lots of material and complete barnacle shells measured up to 6.5 cm in height. No opercular plates (terga or scuta) were visible inside any barnacle shells and the isolated opercular plates were mostly small. Both shells and opercular plates exhibit high degrees of abrasion. Additionally, some of the larger barnacles have small calcite crystals between the inner and outer walls of their abraded shells. Further preparation of matrix-filled shells may yield associated opercular plates which will allow for confident identification. Tentatively, the larger barnacles are placed in the genus *Tamiosoma*. Most suspension-feeding acorn barnacles inhabit shallow water environments attached to hard substrates (e.g., rocks, mollusk shells). Other common arthropod fossils found in the Alajuela Formation include several species of the infaunal, deposit-feeding mud shrimp (Family Callianassidae). Remains include isolated fingers and rarer propodi picked from sediments collected at localities 630041, 630043, 630048, and 630049. As the common name suggests, callianassid shrimp typically inhabit burrows in muddy to sandy bottom intertidal to occasionally subtidal marine habitats.

#### Phylum echinodermata

Remains of fossil echinoderms are rare in the Alajuela Formation exposed on the shores of Lago Alajuela and mostly consist of fragmented radioles (spines) from one (or more?) species of irregular (infaunal) echinoid. All were picked from sediments derived at two localities, 630048 and 630053. However, three regular (epifaunal) echinoid radioles (UF-I 261119) were also collected from locality 630053 and are tentatively identified as belonging to the genus *Prionocidaris*. Additionally two echinoid tests, one a small (< 1 cm), poorly preserved, unidentifiable sand dollar (UF-I 261085) from locality 630043 and one a large (13.5 cm), exceptionally preserved test of the genus *Clypeaster* (UF-I 261132) from locality 630061 were collected. The *Clypeaster* closely resembles *Clypeaster gatuni* Jackson, 1917, from the Gatun Formation, in shape (length and width) but the Alajuela Formation specimen is much more inflated. Today, *Clypeaster* occurs in shallow-water, tropical marine environments.

## Vertebrate Fauna

### Chondrichthyes

**Fig 9:**
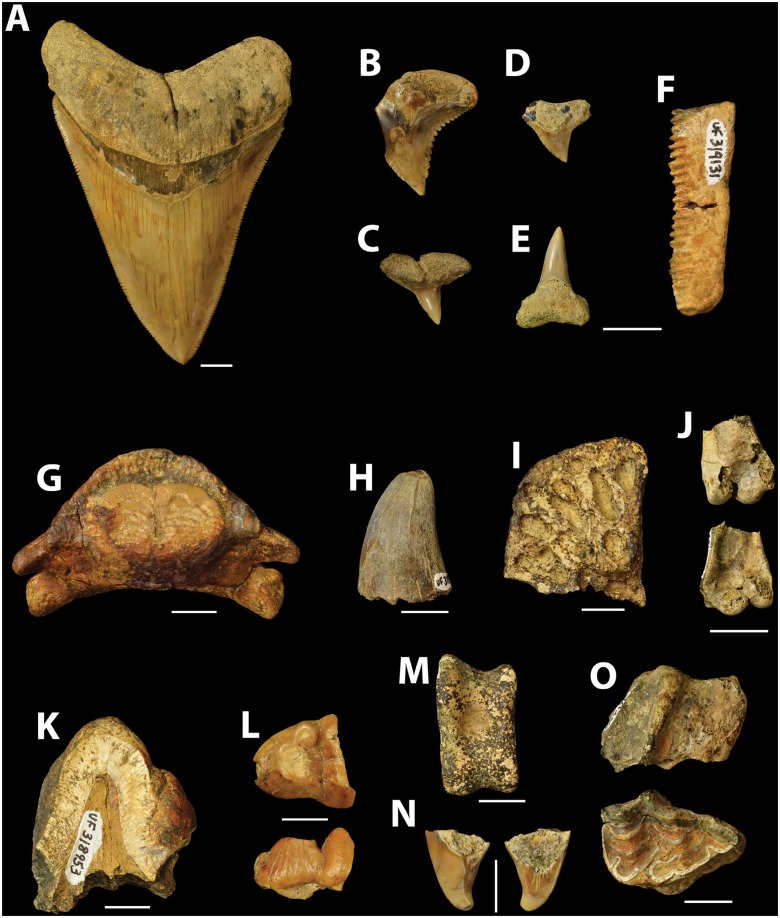
Representative examples of vertebrate fossils collected from the Alajuela Formation, Lago Alajuela, Panama. Most of these fossils were collected from Interval 1, within a zone bracketed by the Sr-ratio age determination (Fig 3). A. UF-V 318900, *Carcharocles megalodon*, right upper anterior tooth, lingual view. B. UF-V 319140, *Hemipristis serra*, right upper tooth, lingual view. C. UF-V 319139, *Sphyrna mokarran*, tooth, lingual view. D. UF-V 318917, *Carcharhinus* sp., right upper tooth, lingual view. E. UF-V 318927, *Negaprion brevirostris*, lower tooth, lingual view. F. UF-V 319131, Myliobatidae, partial tooth, ventral view. G. UF-V 318943, Diodontidae, right and left fused dentaries, dorsal view. H. UF-V 318945, Crocodylia, tooth, anterior or posterior view. I. UF-V 318949, Crocodylia, partial osteoderm, dorsal view. J. UF-V 318954, Aves, distal left humerus, medial (top) and lateral (bottom) views. K. UF-V 318953, *Gomphotherium* sp., tooth fragment. L. UF-V 403570, Gomphotherium sp., anterior portion of left dp2, occlusal (top) and buccal (bottom) views. M. UF-V 318955, Tayassuidae, right astragalus, anterior view. N. UF-V 403571, Carnivora, left I3, distal (left) and mesial (right) views. O. UF-V 321263, *Dinohippus* sp., right upper P2, buccal (top) and occlusal (bottom) views. Scale bars = 1 cm. The testudines (turtles and tortoises) are not included here because they are being described in another paper [52].

So far, 42 chondrichthyan teeth have been collected from the shorelines and islands of Lago Alajuela, representing five different families: Otodontidae, Carcharhinidae, Sphyrnidae, Pristidae, and Myliobatidae (see representative examples in Fig 9). By no means is this a comprehensive collection of all taxa likely present; however, the relative abundances of the specimens known thus far are as follows: The family Otodontidae is represented by a single species, *Carcharocles megalodon*. It is the most abundant taxon (N = 15) and likely reflects collection bias towards larger, more visible fossils seen during surface prospecting. Carcharhinidae is the most speciose family, consisting of *Carcharhinus* spp. (N = 7), *Negaprion brevirostris* (N = 6), and *Hemipristis serra* (N = 2). Sphyrnidae is represented by a single specimen attributed to *Sphyrna mokarran* based on its moderate size, deep distal notch, and serrated cutting edges. The family Myliobatidae is represented by 10 fragmentary teeth, most of which can likely be attributed to *Myliobatis* sp. or *Rhinoptera* sp. Hovestadt and Hovestadt-Euler [43] proposed that intergeneric differences within Myliobatinae, in particular the locking mechanism between teeth, enable distinction between genera; however interspecific variability within genera, such as *Myliobatis*, may be too great to warrant species-level identifications. Similarly, Cappetta [44] noted significant variations between species within the genus *Rhinoptera*. Consequently, species-level identifications were not justified for teeth attributed to Myliobatidae; and given the fragmentary preservation of the teeth, generic assignments may require more rigorous comparisons with other fossil and living representatives.

Among the eight taxa recognized, *C*. *megalodon* and *H*. *serra* are extinct, but both have a cosmopolitan distribution in the fossil record [2]. *Hemipristis serra* is especially common in neritic, warm-water environments during the Miocene and Pliocene [44]. *Negaprion brevirostris* and *Sphyrna mokarran* are extant taxa with a circumtropical distribution [45]. Both taxa are often associated with reef environments and generally populate waters less than 100 m depth [45, 46]. *Carcharhinus* spp. are the most abundant sharks in modern nearshore environments [47, 48] and occur in all temperate and tropical seas [44]. A single rostral denticle was collected by John Turner in 1959. Its flattened conical shape is diagnostic of the sawfish *Pristis* sp., which is a genus with a widespread fossil record during the Cenozoic [7]. *Pristis* sp. also has a cosmopolitan distribution today and inhabits marine tropical and subtropical waters, but may enter freshwater and estuarine environments as well [49, 50]. Within the Neotropics, *Pristis* sp. has been documented from the fossil record of Venezuela, Costa Rica, and Panama [51]. Genera within the family Myliobatidae are often found in tropical to warm temperate seas, especially in shallow waters [43]. All taxa indicate a tropical, shallow water environment; however additional collecting must be undertaken in the future to gain a more holistic perspective of the chondrichthyan fauna of Lago Alajuela.

### Testudines

Turtle shell fragments are frequently encountered in outcrops of the Alajuela Formation, although many of them are difficult to identify below the family level because of the lack of diagnostic characters in the preserved material. These fossils, which were recently reviewed by Bourque et al. [52], consist of at least four taxa (Table 3). In the exception to the rule in terms of fossilization, Bourque et al. [52] describe a well-preserved partial skull of a side-necked turtle that they refer to a new species of the pleurodire *Bairdemys*. The reminder of the testudine samples are represented by cryptodires, including soft-shelled turtles (Trionychidae), tortoises (Testudinidae), and sea turtles (Chelonia). Although not a taxonomically diagnostic collection, the paleoecological implications for the presence of these four testudinid groups will be described below.

**Table 3:** Preliminary list of fossils collected from the Alajuela Formation, late Miocene of Panama.

	**Plants (Angiosperms)**		
Arecales			
	Arecaceae (Palm family)		
		*Palmoxylon* sp. A	
		*Palmoxylon* sp. B	
Fabales			
	Fabaceae (Legume family)		
		gen. et sp. indet.	
Magnoliales			
	Annonaceae (Custard apple family)		
		*Annonoxylon* sp.	
Malpighiales			
	Chrysobalanaceae		
		*Parinarioxylon* sp.	
	Humiriaceae		
		*Humiriaceoxylon* sp.	
	?Phyllanthaceae		
		gen. et sp. indet.	
		(cf. Achariaceae, Malpighiaceae)	
Malvales			
	Malvaceae (Mallow family)		
		gen. et sp. indet.	
Sapindales			
	Sapindaceae (Soapberry family)		
		gen. et sp. indet.	
	**Invertebrata**		
Brachiopoda			
	Lingulidae		
		*Glottidia* sp.	
Bryozoa			
	Cheilostomata		
		Chapperiidae	
			*Chaperia* sp.
		Hippoporidridae	
			*Hippoporidra* sp.
		Family indet.	
	Cyclostamata		
		Plagioeciidae	
			gen. et sp. indet.
		Family indet.	
Annelida			
	Polychaeta		
		Family indet.	
Mollusca			
	Bivalvia		
		Yoldiidae	
		Arcidae	
		Pectinidae	
			*Euvola* sp.
			*Nodipecten* sp.
		Plicatulidae	
			*Plicatula* sp.
		Anomiidae	
			*Anomia* sp.
		Flemingostreidae	
			*Crassostrea* sp.
		Cardiidae	
			*Venericardia* sp.
		Pharidae	
			*Ensis* sp.
		Tellinidae	
		Veneridae	
			*Chione* sp.
			*Chionopsis* sp.
			*Lirophora* sp.
			*Macrocallista* sp.
			*Pitar* sp.
		Corbulidae	
		Gastrochaenidae	
		Hiatellidae	
			*Panopea* sp.
	Scaphopoda		
		Family indet.	
	Gastropoda		
		Cerithiidae	
		Turritellidae	
		Strombidae	
			*Strombus* sp.
		Xenophoridae	
			*Xenophora* sp.
		Calyptraeidae	
			*Calyptraea* sp.
		Cypraeidae	
		Naticidae	
		Tonnidae	
			*Malea* sp.
		Buccinidae	
			*Cymatophos* sp.
		Melongenidae	
			*Melongena* sp.
		Turbinellidae	
			*Turbinella* sp.
		Olivellidae	
			*Olivella* sp.
		Olividae	
			*Oliva* sp.
		Marginellidae	
		Conidae	
			*Conus* sp.
		Terebridae	
			*Terebra* sp.
		Turridae	
		Architectonicidae	
		Haminoeidae	
	Cephalopoda		
		Coleoidea	
Arthropoda			
	Malacostraca		
		Callianassidae	
			gen. et sp. undet.
	Cirripedia		
		Balanidae	
			cf. *Tamiosoma* sp.
Echinodermata			
	Echinoidea		
		Cidaridae	
			cf. *Prinocidaris* sp.
		Clypeasteridae	
			*Clypeaster* sp.
	**Vertebrata**		
Chondrichthyes			
	Lamniformes		
		Otodontidae	
			*Carcharocles megalodon*
	Carcharhiniformes		
		Carcharhinidae	
			*Hemipristis serra*
			*Carcharhinus* spp.
			*Negaprion brevirostris*
		Sphyrnidae	
			*Sphyrna mokarran*
	Pristiformes		
		Pristidae	
			*Pristis* sp.
	Myliobatiformes		
		Myliobatidae	
			*Myliobatis* sp.
			*Rhinoptera* sp.
Reptilia			
	Testudines*		
		Podocnemididae	
			*Bairdemys* n. sp.
		Trionychidae	
			gen. et sp. indet.
		Testudinidae	
			gen. et sp. indet.
		Cheloniidae	
			gen. et sp. indet.
	Crocodylia (longirostrine)		
		Tomistominae	
Aves			
	gen. et sp. indet.		
Mammalia			
	cf. Carnivora		
	Sirenia		
		cf. Dugongidae	
	Proboscidea		
		Gomphotheriidae	
			*Gomphotherium* sp.
	Artiodactyla		
		Tayassuidae	
			gen. et sp. indet.
	Perissodactlya		
		Equidae	
			*Cormohipparion* sp.
			*Dinohippus* sp.

*From Bourque et al. [52].

### Crocodylia

Isolated crocodylian elements were collected from several localities along the shores of Lago Alajuela. Crocodilian fossils come from Interval 1, most of them were collected in the fine and medium grained sandstones, whereas others were found in amalgamated conglomerate lenses. Isolated vertebrae have fused neurocentral sutures [53] and represent mature individuals. Their size is comparable to that of living crocodilians between 3–4 m long. Most osteoderms, although incomplete, preserve straight margins and well defined corners, indicating that their shape was roughly square. They also lack keels on their external surfaces. Crocodilian teeth are represented by two morphotypes, i.e., a slender, sharp morphotype, and blunt, robust one.

The most diagnostic elements recovered so far are an almost complete axis and partial maxilla. Both have features consistent with longirostrine crocodilians. However, they lack key features diagnostic of living *Gavialis* and South American gavialoids. These remains probably represent tomistomines, likely related to the North American genus *Thecachampsa* (= *Gavialosuchus*). Crocodylian fossils attributed to *Thecachampsa* have been collected in the upper Miocene Curré Formation of Costa Rica [54]. Longirostrine crocodylians from the early Miocene of the Panama Canal basin could also be related to *Thecachampsa* [55].

### Aves

A single fragmentary left distal humerus, UF-V 318954 (Fig 9), although identifiable as a bird, is otherwise not taxonomically diagnostic (Steadman, personal communication, 2015).

### Mammalia

#### Cf. carnivora

A single left I3, UF-V 403571, is attributable to a carnivoran, but is not diagnostic at a lower taxonomic level.

#### Cf. dugongidae

Several rib fragments have been collected that are otherwise undiagnostic except for likely being from a sirenian based on the oval cross-section and internal pachyostotic condition characteristic of these marine mammals. It is tempting to speculate that these might pertain to the cosmopolitan Miocene genus *Metaxytherium*, but this is not warranted until more diagnostic material is collected because there are several endemic Caribbean extinct dugongids [56].

#### Proboscidea

The genus *Gomphotherium* was recently described from Lago Alajuela [6] based on a diagnostic 1^st^ lower molar, UF-V 294322. In July 2015 we confirmed with the collector, John Turner, where the tooth was discovered in the field in 1959; the approximate location (between 630042 to 630049) also pertains to Interval 1 in our composite stratigraphic section (Fig 3). An additional specimen, represented by a left dp2 (UF V 403570), has been recovered from Isla Vigia (Table 1, 630050). Other proboscidean tooth fragments that likely pertain to this genus have been collected (Fig 9) from several of our localities, but primarily from the lower part of the section (Interval 1). *Gomphotherium* has a relatively long biochronological range in North America from about 15 to 5 Ma. In the absence of other chronological information and the lack of a definitive species determination for this genus, we were unable to make a more precise age determination for the Alajuela Formation. But, with the new Sr-ratio age determinations reported here, the occurrence of *Gomphotherium* in Panama has significance with regard to previous hypotheses concerning the timing of dispersal into Central America during the Miocene.

#### Cf. tayassuidae

A single right astragalus, UF-V 318955 (Fig 9), indicates the presence of a medium-sized artiodactyl that likely represents a tayassuid based on our comparisons with other specimens of Miocene peccaries in the UF-V collection.

#### Equidae

Two specimens of equids have been collected, including (1) a right P2 (UF-V 321263) of cf. *Dinohippus* sp. (Fig 9), likely referable to this genus based on tooth size, simple enamel pattern, and the strength of the connection of the protocone to the protoselene; and (2) a partial upper molar fragment (UF-V 321264) likely referable to *Cormohipparion* sp. based on tooth size and the complexity of enamel plications.

## Discussion and Significance

### Biochronology and Age Assignment

All of the taxa collected from Lago Alajuela so far have only been identified to the genus or higher taxonomic level, with the exception of some of the species of sharks. We anticipate that as more fossils are collected of temporally diagnostic taxa and additional detailed studies of individual groups are undertaken in the future, some of the fossils collected from Lago Alajuela will be identified to species. As such, these fossils may then provide a more precise biochronological assessment of the Alajuela Formation.

The invertebrate taxa represent genera that are long-lived and are known to have occurred during the Neogene. Likewise, the fossil plants found adjacent to Lago Alajuela are referred to modern families. With regard to the vertebrates, the sharks and reptiles likewise have long temporal ranges, and as such, are not particularly useful biochronologically, other than also being late Cenozoic or Neogene, similar to the invertebrates. With regard to the mammals, the proboscidean *Gomphotherium* is known to have occurred elsewhere in North America during the middle to late Miocene and early Pliocene, from ca. 15 to 5 Ma [6]. Despite its relatively long range in higher-latitude North America, Lucas and Alvarado [57] assert that *Gomphotherium* did not disperse into lower latitudes, i.e., Central America, until the late Hemphillian. The two equid genera have late Miocene to early Pliocene temporal ranges, and are similar to those forms previously described from Costa Rica [58] that are referred to the Hemphillian North American Land Mammal Age, which spans an interval from 9 to 4.8 Ma in higher-latitude North America [59].

Given the current state of our knowledge about the fossil faunas and floras from Lago Alajuela, the biochronological information suggests a late Miocene age for the Alajuela Formation. We are hopeful that we might find diagnostic and biogeographically interesting microfossils, e.g., foraminifera and mammals such as rodents, bats, or possibly even primates (e.g., Bloch et al. [60]) through screen-washing and sieving, but up to now we have been unsuccessful in this regard. As we continue to process and sort screenwashed matrix, the potential for adding to the vertebrate fauna remains a distinct possibility.

As such, we must turn to the Sr-ratio ages derived from *in situ* invertebrate fossils to provide additional clarity in terms of the age of the Alajuela Formation. It should be noted here that most of the diagnostic mammal taxa are collected from the same zone as the Sr-ratio ages (Fig 3). The mean age of 9.77 ± 0.22 Ma (Table 1) places our measured section within the late Miocene, and latest Clarendonian, not Hemphillian, NALMA (Cl3, *sensu* [59]). A close temporal equivalent in higher latitude North America is represented by the Love Bone Bed Local Fauna of north-central Florida [61].

### Paleoecology

Taken together, the plant, invertebrate, and vertebrate fossils from the Alajuela Formation are diagnostic in terms of reconstructing the paleoenvironments and paleoecology during the late Miocene in this region of what is now Panama. The sharks, sirenians, and some of the invertebrates (e.g., *Clypeaster*) indicate a shallow-marine environment. The land mammals, other invertebrates (e.g., *Crassostrea*), and ichnofossils indicate an estuarine environment with local influence from terrestrial environments. Terrestrial vertebrate fossils include the proboscidean, horses, peccary, carnivoran, and tortoise. The trionychid turtle indicates freshwater habitats. The crocodilians and some of the other turtles confirm the presence of transitional estuarine and shallow marine environments.

We understand that the silicified woods in the Alajuela Formation are likely reworked, and are unsure how much older these fossils are relative to the in situ invertebrates and vertebrates. Regardless, all of these wood families are common in tropical forests in Panama today [21, 22], and from an analysis of their anatomy indicate the presence of large trees. This evidence thus speaks to a temporal duration from the early Miocene to the Recent in which these habitat-types are represented in Panama.

### Late Miocene Seaway in Panama

Although traditionally it has been argued that the Central America Seaway functioned until the late Miocene, with final closure during the Pliocene at about 4 to 5 Ma, recent evidence argues for an earlier closure. Montes et al. [40] suggest that closure of the seaway occurred significantly earlier, i.e., during the middle Miocene, ca. 13 to 15 Ma. The younger evidence from Alajuela as well as from roughly contemporaneous localities from the Gatun [1] and Chucunaque [62] formations in Panama speak to this debate.

The abundance of marine fossils from the Alajuela and Gatun formations, and as shown by Coates et al. [5] for the Chucunaque Formation, demonstrates seaway connections existed through central Panama during the late Miocene. Strontium ratios also suggest the Alajuela Formation overlaps in time with the richly fossiliferous upper Miocene Gatun Formation to the north with which it shares many invertebrate faunal elements. Preliminary interpretations suggest the Alajuela Formation represents a higher energy, near-shore, marine setting that episodically received riverine/terrestrial input and was perhaps shallower and more proximal to a coastline than either the Chucunaque or Gatun formations. Future work will help to elucidate these important relationships.

## Concluding Comments

The discovery of a new fossil assemblage of plant, invertebrate, and vertebrate fossils from the Alajuela Formation at Lago Alajuela has the potential to advance our understanding of the evolution of tropical marginal marine and terrestrial ecosystems in Central America, as well as further address the current debate about the formation of the isthmus. The calibration of this assemblage using Sr-isotope ratio dating to 9.77 Ma ± 0.22 Ma provides precise temporal control otherwise unknown for the late Miocene in Central America. This indicates contemporaneity with the Gatun and Chucunaque formations in Panama as well as a late Clarendonian (Cl3) North American Land Mammal age as this biochron is otherwise known in higher latitude North America. We understand that we have not yet sampled the full range of ancient diversity at Lago Alajuela; we hope that further discoveries will be forthcoming with additional field work. Likewise, the taxonomic assignments that we present here will likely become more refined as individual taxonomic groups are compared in more detail in the future.

## Supporting Information

S1 Table Analysis and comparisons of fossil woods studied. (XLSX)

S2 Table Panama REE (rare earth element) data analyzed in? 2015. (XLSX)
